# Science in the Courtroom: Examining Standards for Litigation-Based Research

**DOI:** 10.1289/ehp.116-a37

**Published:** 2008-01

**Authors:** Richard C. Dahl

Over the last 20 years, the term “junk science” has gained increasing use by defendants in toxic tort litigation as a pejorative phrase to discredit health effects data that do not meet some standard for scientific validity—or, some say, that are favorable to the interests of plaintiffs. Proponents of tort reform have argued that many large jury verdicts are the unjustified products of questionable scientific data presented by plaintiff lawyers to easily swayed jurors. Courts have responded by raising the bar that scientific evidence must exceed in order to be admitted as evidence. But has this change produced sound results? Is there really a distinction between litigation-based science and other science? In a mini-monograph in this issue, 5 articles examine these questions and others that arise when examining the juncture of science and litigation **[*EHP* 116:116–147]**.

Acknowledging that conflicts of interest are an inherent component of science-based litigation, authors Ronald L. Melnick, Kristina A. Thayer, and John R. Bucher of the NIEHS conclude that public health decisions to allow exposure to possible carcinogens should not rely “on untested hypotheses that are promoted to explain away adverse outcomes.” Their article focuses specifically on rodent carcinogenicity studies and examines how strict attention to design and evaluation can reduce inaccurate conclusions and provide data that are useful for evaluating human health risks.

The authors cite early animal studies on benzene as an example of poor design that failed to detect carcinogenic effects, even though epidemiologic studies demonstrated a causal association between benzene exposure and leukemia in humans. Those early studies employed too few animals, insufficient controls, too short a study duration, and inadequate levels of exposure. In addition, the authors write, “evaluations that are based on incomplete necropsy or histopathology, do not combine related tumor effects, fail to adjust for differences in animal survival, or incorrectly use historical control data would not be expected to produce reliable information on chemical carcinogenesis.”

Courts, meanwhile, have also taken steps to reduce the likelihood of “junk science” influencing juries. Two of the articles in the mini-monograph, the first by Leslie I. Boden and David Ozonoff of the Boston University School of Public Health and the other by Sheila Jasanoff of Harvard University’s Kennedy School of Government, examine the assertion that science conducted to support litigation must be held to tougher admissibility standards than other science. Appeals Court Judge Alex Kosinski made this claim in 1995 in response to the Supreme Court remand in *Daubert v. Merrell-Dow Pharmaceuticals, Inc.*, deciding that judges were henceforth required to assume roles as gatekeepers with the responsibility of culling out unreliable expert evidence. As the authors describe, many courts following *Daubert* have held that research conducted specifically for the purpose of a litigation is inherently less reliable than other science.

Boden and Ozonoff re-examine whether litigation-based science should be treated differently from other science offered as evidence in the courtroom. They conclude that it shouldn’t. Their contentions include an assertion that cross-examination by attorneys aided by competent experts, not just journal peer review, also serves the ends of justice. They further argue that any science is subject to a variety of biases; for example, they write, studies funded by pharmaceutical companies or investments by corporations in research agendas tend to favor their own economic interest. Finally, the authors argue that tougher standards for litigation-generated science unfairly burden plaintiffs.

In her article, Jasanoff agrees that restrictions placed on litigation-based science following *Daubert* are misconceived because the scientific knowledge needed to resolve legal disputes often arises only in response to litigation. Rather than assign judges the role of gatekeeper, a more sensible approach, she writes, would be for judges to assume the position of referee. In this role, judges would “focus on the process through which litigation science is generated rather than on its validity or invalidity. They would be in a position to structure agreements among the parties that would be most conducive to producing relevant and reliable knowledge.”

In a fourth article, Carol J. Henry and James W. Conrad, Jr., of the American Chemistry Council focus on the role of federal agencies rather than that of the courts. They write that the quality of agency scientific research and testing is already subject to a variety of standards and practices (e.g., the Federal Information Quality Act, and peer review and transparency in research practices), and argue that these standards and practices allow agencies to judge the quality of work regardless of the reason for which it was created. They also point out that federal agencies are required to accept and fairly consider information provided by any interested person in the course of decision making.

In the last paper, William R. Freudenburg of the University of California, Santa Barbara, takes a critical look at the nature of bias itself, concluding that scientists oftentimes are not conscious of its influence on them. Drawing from personal experience, Freudenburg describes litigation-based research he conducted for a company that never tried to censor his work and consistently praised him for being principled and credible. But he subsequently came to realize that praise for his objectivity actually encouraged him to interpret his findings in ways that would favor his corporate sponsors more than if they had tried to tell him what to say. The problem, he writes, was “the temptation to start changing my own judgments . . . in response to their repeated insistence that it was precisely my independent and scientific credibility that they valued.”

The articles in the mini-monograph share a common thread: when science is used to serve the purposes of litigation or administrative proceedings, great care is needed to ensure its proper deployment, and a courtroom judge is probably not the appropriate person to decide on the reliability and relevance of scientific evidence. Furthermore, the perception that bias is inherently bad or avoidable may itself be biased.

## Figures and Tables

**Figure:**
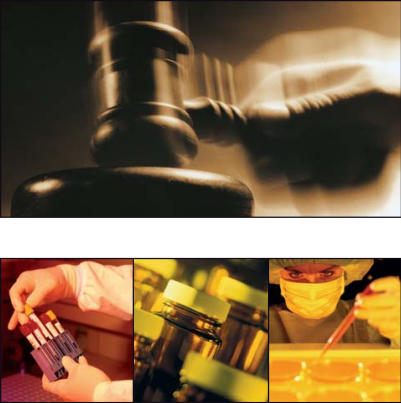
Should research conducted expressly for court use be held to higher standards than any other research?

